# Inferring Endozoochory From Ingestion to Germination Through Biological Filters: Brown Bear Feces as a Case Study

**DOI:** 10.1002/ece3.72589

**Published:** 2025-12-02

**Authors:** Grégoire Pauly, Cécile Vanpé, Mélanie Roy, Yannis Clavier, Adélie Chevalier, Jérôme Sentilles, Tanguy Daufresne, Christophe Baltzinger

**Affiliations:** ^1^ INRAE, UR 1455 EFNO Nogent‐sur‐Vernisson France; ^2^ UMR Eco&Sols, INRAE Montpellier Cedex 2 France; ^3^ Office Français de la Biodiversité (OFB), Service conservation et gestion des espèces à enjeux Villeneuve de Rivière France; ^4^ UMR CRBE, Université de Toulouse Toulouse Cedex 9 France; ^5^ Instituto Franco‐Argentino para el Estudio del Clima y sus Impactos (UMI IFAECI/CNRS‐CONICET‐UBA‐IRD) Universidad de Buenos Aires Buenos Aires Argentina; ^6^ UMR 5175 CEFE Montpellier 5 France

**Keywords:** ferns, germination, metabarcoding, mosses, seed dispersal, viability test

## Abstract

Diaspore (e.g., seed and spore) dispersal is recognized as a key mechanism in plant dynamics, including endozoochory, which can be a risky journey for diaspores. Endozoochory is achieved when diaspores are consumed and may germinate after the mastication, the gut and fecal matrix passage, all representing filters for diaspores. Nevertheless, endozoochory is a highly studied mechanism through numerous methods, notably based on the observation of frugivorous behavior, diaspores retrieved in feces or germination experiments. However, most of those methods consider partially the endozoochorous filters (ingestion, mastication, gut, feces). Hence, the combined effect of the methods and filters consideration may lead to biased inference of endozoochory. In this study, we used a collection of 52 brown bear ( *Ursus arctos* ) feces to highlight five methods inferring endozoochory. Two methods consider the ingestion filter and used metabarcoding of fecal eDNA to identify fleshy fruits (i) or plants during fruiting periods (ii). The third method (iii) was based on the intact propagules retrieved in feces, considering ingestion and mastication filters. Another method (iv) was based on the germination from disaggregated feces, considering up to the gut passage filter. The last method (v) was based on the germination from aggregated feces, considering the four filters. We compared the number of taxa, the community and the plant life forms inferred among methods. We inferred the largest number of taxa in method (iii), but the germination‐based methods inferred the most diverse plant life forms. We identify few shared taxa among methods. The metabarcoding‐based methods might be an interesting tool as a first approximation of endozoochory while detailing the diet. The method (v) appeared as the most reliable. Overall, we invite future studies to mitigate their interpretations according to the filters of endozoochory considered and plant detectability related to the method used.

## Graphical abstract

Our study investigated the combined effect of methods and endozoochorous filters (ingestion, mastication, gut passage and fecal matrix) on the inference of endozoochory. Our results highlighted differences according to the method used on the inference of endozoochory in terms of number of taxa and plant life forms, while inducing variable reliability based on the endozoochorous filters considered. Our study invites future research to mitigate the interpretation of endozoochory according to the filters considered and the method used.

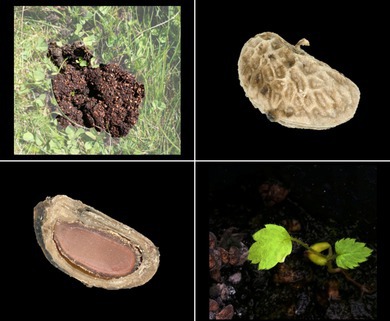

## 1 Introduction

Plant population dynamics in ecosystems are determined by many biotic and abiotic mechanisms, among which diaspore (e.g., seed or spore) dispersal across the landscape is crucial (Beckman and Sullivan 2023). Indeed, diaspore dispersal is a key mechanism for colonization or escape from harsh environments due to competition, predation, or pathogens. Diaspores can be dispersed by various vectors, either abiotic (e.g., the wind, anemochory) or biotic (i.e., zoochory) (Howe and Smallwood 1982). For instance, vertebrates may transport diaspores over long distances by epizoochory through adhesion to their fur, hoof or feathers or endozoochory through ingestion and excretion by defecation or regurgitation (e.g., Baltzinger et al. 2019).

Endozoochory by vertebrates has received considerable attention in past decades (e.g., Nogales et al. 2024) and if it was related to frugivores and fleshy fruit ingestion in the first place (i.e., endozoochorous dispersal syndrome, Van der Pijl 1982), other guilds and plant types are now documented as involved in this dispersal mechanism. In particular, large herbivores (Baltzinger et al. 2019) and omnivores (García‐Rodríguez, Albrecht, et al. 2021) have been well studied, and numerous pieces of evidence exist for the dispersal of dry‐fruited plants (Lalleroni et al. 2017; Pauly et al. 2024), ferns (Lovas‐Kiss et al. 2018; Pauly et al. 2024) or mosses (Parsons et al. 2007; Pauly et al. 2024). However, endozoochory has been studied and inferred in many different ways, either based on frugivorous behavior, diaspores retrieved in feces, or germination experiments, as shown in a review on islands (Nogales et al. 2024), seed dispersal networks (Dáttilo 2025) or palms (Marques Dracxler and Kissling 2022). Similar observations can be made if we focus on a given species, such as the brown bear ( *Ursus arctos* ), for which endozoochory was inferred either by monitoring diaspores retrieved in feces (Lalleroni et al. 2017), germination of diaspores extracted from feces (Tavşanoğlu et al. 2021), or germination experiments using intact feces (Pauly et al. 2024). And yet, although all these studies deal with endozoochory, we can legitimately wonder whether they all provide a reliable inference of this complex mechanism (Dáttilo 2025).

Endozoochory is a risky and hazardous process for the plant diaspores, which pass through at least four successive filters to be dispersed (i.e., described hereafter), and numerous methods consider only partially these filters. For instance, the first filter is the effective ingestion of diaspores, which can be inferred by visual observation of frugivory (e.g., Harrer and Levi 2018) or through eDNA fecal metabarcoding to detect fleshy fruits (endozoochorous syndrome, e.g., Sato et al. 2025). Once eaten, diaspores are often masticated (i.e., second filter) and potentially broken, drawing the limit between diaspore dispersal and predation (Janzen 1971). The study of intact diaspores retrieved from feces enables a consideration of this filter (Lalleroni et al. 2017). Subsequently, the diaspores are within the gut (i.e., third filter), which may cause an abrasive detrimental effect on the diaspore or enhance their viability through the action of digestive acids (Traveset and Verdú 2002). Studies testing diaspore viability (e.g., using Tetrazolium, Farmer et al. 2017) or germination experiments of diaspores retrieved in feces (Tavşanoğlu et al. 2021; Tsunamoto et al. 2024) properly consider the filters up to the gut passage. Finally, when the animal defecates, the diaspores can be constrained for germination by the fecal matrix through chemical or mechanical constraints (Mancilla‐Leytón et al. 2012; Pauly et al. 2024; Jiménez‐Martín et al. 2025), and germination experiments based on intact feces permit assessing this filter (e.g., Mancilla‐Leytón et al. 2012; Pauly et al. 2024; Jiménez‐Martín et al. 2025). In addition, germination and establishment stages also depend on the quality of the releasing site (Schupp et al. 2010; Karimi et al. 2020; García‐Rodríguez, Selva, et al. 2021) and on secondary dispersal events (Ishikawa 2011; Culot et al. 2015; Pauly, Vanpé, Roy, Sentilles, et al. 2025). Nevertheless, according to these four filters (ingestion, mastication, gut passage, and feces), a partial consideration of at least one of them in the study of endozoochory may lead to biased results and conclusions.

Typically, the use of different methods to infer endozoochory complicates comparisons among studies, but we argue that it is exacerbated by the variability in the inference reliability according to the endozoochorous filters considered (Dáttilo 2025). For instance, several studies based on target plants and using captive animals, highlighted more intact diaspores (up to the mastication filter) than germinated diaspores (up to the gut passage filter) (Mancilla‐Leytón et al. 2011; Grande et al. 2013; Rubalcava‐Castillo et al. 2023; Tsunamoto et al. 2024), illustrating the inference variability according to the filters considered. In this regard, the combined effect of the method and the filters considered can lead to biased results, with varying degrees of confidence. Both can affect particularly the results of the number of plant taxa retrieved and the type of plant life form and dispersal syndrome inferred. For the latter, it is particularly important, as dispersal syndromes are not fully reliable for endozoochory, especially for omnivores and herbivores (Green et al. 2022). In this context, the investigation of endozoochory inference according to different methods could be particularly relevant.

Therefore, we investigated the inference of endozoochory according to five different methods that consider part or all of the four endozoochorous filters (i.e., ingestion, mastication, gut passage, fecal matrix). To this purpose, we used 52 brown bear feces collected in the Pyrénées (South‐West Europe), a potential keystone species (Pauly, Vanpé, Roy, Quenette, et al. 2025) and a well‐known plant disperser (García‐Rodríguez, Albrecht, et al. 2021) of different plant life‐forms (Pauly et al. 2024). We performed two methods based on fecal eDNA metabarcoding that consider only the ingestion filter, a first one (i) relying on the ingestion of plants with a fleshy fruit syndrome, and a second one (ii) relying on all plants ingested during their fruiting periods (i.e., propagules ingested). We performed a third method (iii) that considers up to the mastication filter, based on intact diaspores retrieved from the feces. The fourth method (iv) was based on a germination experiment from disaggregated feces, a method that considers up to the gut passage filter. We additionally used a Tetrazolium test as an alternative to germination experiments. Finally, we inferred endozoochory using a fifth method (v) based on germination experiments from intact and aggregated feces that consider all four filters of the endozoochory process. For each method, we investigated the number of taxa and plant life forms inferred. Furthermore, as our study was based on a comparison of methods, we also provided information related to their applicability. We hypothesized a different number of taxa retrieved among methods and predicted that methods considering only the ingestion filter would provide the largest number of taxa. We also hypothesized that most of the taxa are shared between at least two methods. Finally, we hypothesized that methods based on germination (iv and v) and metabarcoding (only ii) will enable the inference of cryptic taxa dispersed as very small diaspores or spores, which can be missed through visual observation (method iii).

## 2 Materials and Methods

### 2.1 Feces Collection

We collected 52 fresh (less than 48 h‐old) brown bear feces between 2017 and 2022 in the French part of the Pyrénées (18,000 km^2^) using dogs trained to detect brown bear feces (Sentilles et al. 2021). We searched feces from March to November, corresponding to the activity periods of brown bear (i.e., excluding winter dormancy). We searched feces systematically in identified feeding areas (hosting soft or hard mast plants) and opportunistically following recent brown bear sightings, livestock depredations, or apiary damage. Feces were stocked in a freezer at −18°C. Metabarcoding for methods (i) and (ii) was performed during December 2022. The diaspore extraction for method (iii) was performed in spring 2024. The germination experiments of methods (iv) and (v) were performed simultaneously between April and October 2023, results already being published (Pauly et al. 2024).

### 2.2 Metabarcoding Procedure

The metabarcoding was conducted at the Molecular Biology Microbiology facility of the Centre de Recherche sur la Biodiversité et l'Environnement (CRBE) laboratory in Toulouse (France) in December 2022 using a 15‐g portion extracted from the core of the feces collected in the field or in the laboratory after collection. We used the trnL marker to detect the plants ingested (see Supporting Information for details on the metabarcoding procedure) and a reference sub‐database on the plant species present in the Pyrénées, extracted from GenBank to obtain better assignment accuracy at the species level. The 15‐g portion allowed the identification of the plant composition of the entire feces.

### 2.3 Methods for inferring endozoochory

#### 2.3.1 Method (i): Metabarcoding and the Fleshy‐Fruited Plants Ingested

Metabarcoding provides information on the presence of plants in feces, regardless of the part consumed. Here, based on endozoochorous dispersal syndrome (Van der Pijl 1982), we consider fleshy‐fruited plants as consumed for their fruits. We thus inferred endozoochory based on fleshy‐fruited plant occurrences in the diet using metabarcoding results. This method only considers the ingestion filter. We considered all taxa (species or genera) producing only fleshy fruits.

#### 2.3.2 Method (ii): Metabarcoding and Plants Ingested During Fruiting Periods

Here, we consider all the plants retrieved in feces based on metabarcoding results, and inferred endozoochory for plants ingested during their fruiting period (i.e., diaspore ingestion). This method only considers the ingestion filter. To collect the information of the fruiting period, we used three sources: (1) FLORAPYR (https://atlasflorapyrenaea.eu), a Pyrénées flora database; (2) Tela Botanica (https://www.tela‐botanica.org), a collaborative database managed by French botanists; and (3) Pl@ntNet (https://plantnet.org), a database encompassing fruit observations by observers in the Pyrénées, as a form of citizen science. Based on months of fructification recorded in databases, we determined the main fruiting period for months when the three databases were congruent. Otherwise, we considered the remaining months reported as secondary fruiting periods. We did not perform this approach at the genus level because interspecific variation of fruiting periods can be too wide. Hard mast species of Fagaceae (e.g., *Quercus* sp.) were discarded as their diaspores are generally destroyed by mastication (but see Lalleroni et al. 2017).

#### 2.3.3 Method (iii): Seed Extraction

The third method (iii) inferred endozoochory based on the intact diaspores retrieved in the feces. This method considers the ingestion and mastication filters. We used 5 g of fresh matter per individual feces to extract the intact diaspores, similarly to Lalleroni et al. (2017) who used 5 g of dry matter. We passed each feces through three successive sieves (with a mesh of 4.00, 0.80, and 0.08 mm, respectively) and water washed them to break down the fecal parts, remove macroelements, and collect large diaspores and fruits. We then collected the filtered material to search for smaller diaspores. We dried the filtered material to prevent mold formation and sorted all intact diaspores under a binocular magnifying glass. The diaspores retrieved were identified using reference books (Cappers et al. 2006; Cappers and Bekker 2013) and counted up to 100 (as some fruits, such as *Vaccinium* sp. fruits, may contain an extremely high number of seeds).

#### 2.3.4 Method (iv): Seed Germination From Disaggregated Feces

Here, we inferred endozoochory based on the germinated plants identified using a germination experiment on disaggregated feces. This method considers the ingestion, mastication, and gut passage filters. This method and its results were extracted from a previous study using the same samples (Pauly et al. 2024). We collected 75 g of fresh matter from each feces, which were passed through the same successive sieves as method (iii) and water washed to disaggregate the feces and remove macroelements that were not seeds or fruits. The disaggregated material was then deposited on potting soil (blond sphagnum peat moss and coarse perlite, Klasmann/Dielmann) and a wipe (i.e., cotton gauze for medical use) under the potting soil, in a rectangular pot measuring 13 × 9 × 4.5 cm (length by width by height). We left the pots under a greenhouse for 209 days (from 5 April 2023 to 31 October 2023), with frequent watering. After 121 days, we randomly changed the pots' position to limit the effect of placement. We monitored each sub‐sample every 4 days to map and count the new and dead seedlings. To control for external and potting soil contamination, we veiled the experiment during the *Asteraceae* sp. and *Salicaceae* sp. fruiting periods, and used 20 control pots with potting soil only. These control sub‐samples were placed at the same time and in the same greenhouse as the fecal samples. We annotated the germinated plants to the finest taxonomic level based on morphological criteria (Hanf and Martin 1970; Muller 2013). For some plants whose premature death prevented reliable identification, we assigned a probable identification (mostly based on the cotyledons only). We also inventoried and identified all the mosses and liverworts in each sub‐sample just after the end of the 209‐day monitoring period, because the identification required disruptive extractions of the substrate.

We also performed a viability Tetrazolium test as an alternative to germination experiments on plant taxa extracted with the method (iii). We decided to conduct this test on taxa with at least 50 seeds retrieved to properly estimate the effect of gut passage. On those plants, we randomly selected 50 seeds per taxa during summer 2025 and followed the Tetrazolium test procedure outlined in the dedicated handbook (Miller and Peters 2010).

#### 2.3.5 Method (v): Seed Germination From Aggregated Feces

The fifth method (v) inferred endozoochory based on the germinated plants identified using a germination experiment on aggregated feces. This method considers the ingestion, mastication, gut passage, and feces filters. This method and its results were extracted from a previous study using the same samples (Pauly et al. 2024). We collected another 75 g sample of fresh matter from each feces, which was left intact and naturally aggregated, keeping the structural complexity of the feces. The intact aggregated feces were similarly deposited and monitored as for the fourth method (iv) in the same place, at the same time and with the same protocol.

#### 2.3.6 Comparison of Methods

We compared the plant detectability among methods using accumulation curves based on the number of taxa detected and the number of feces. For this purpose, we removed unidentified taxa or uncertain identification. In a second analysis, we compared plant communities dispersed based on a dissimilarity matrix using a permutation test and by calculating the turnover and nestedness among methods. For this purpose, for a given method that identified a taxon at the species level (e.g., *Rubus idaeus* ), an occurrence at the genus level was recorded if that genus had been identified at that taxonomic rank by another method (e.g., *Rubus*). Finally, we qualitatively described for each of the methods the ability to detect different plant life forms, and applicability related to the time, expense (except agent salary), and expertise required.

**FIGURE 1:**
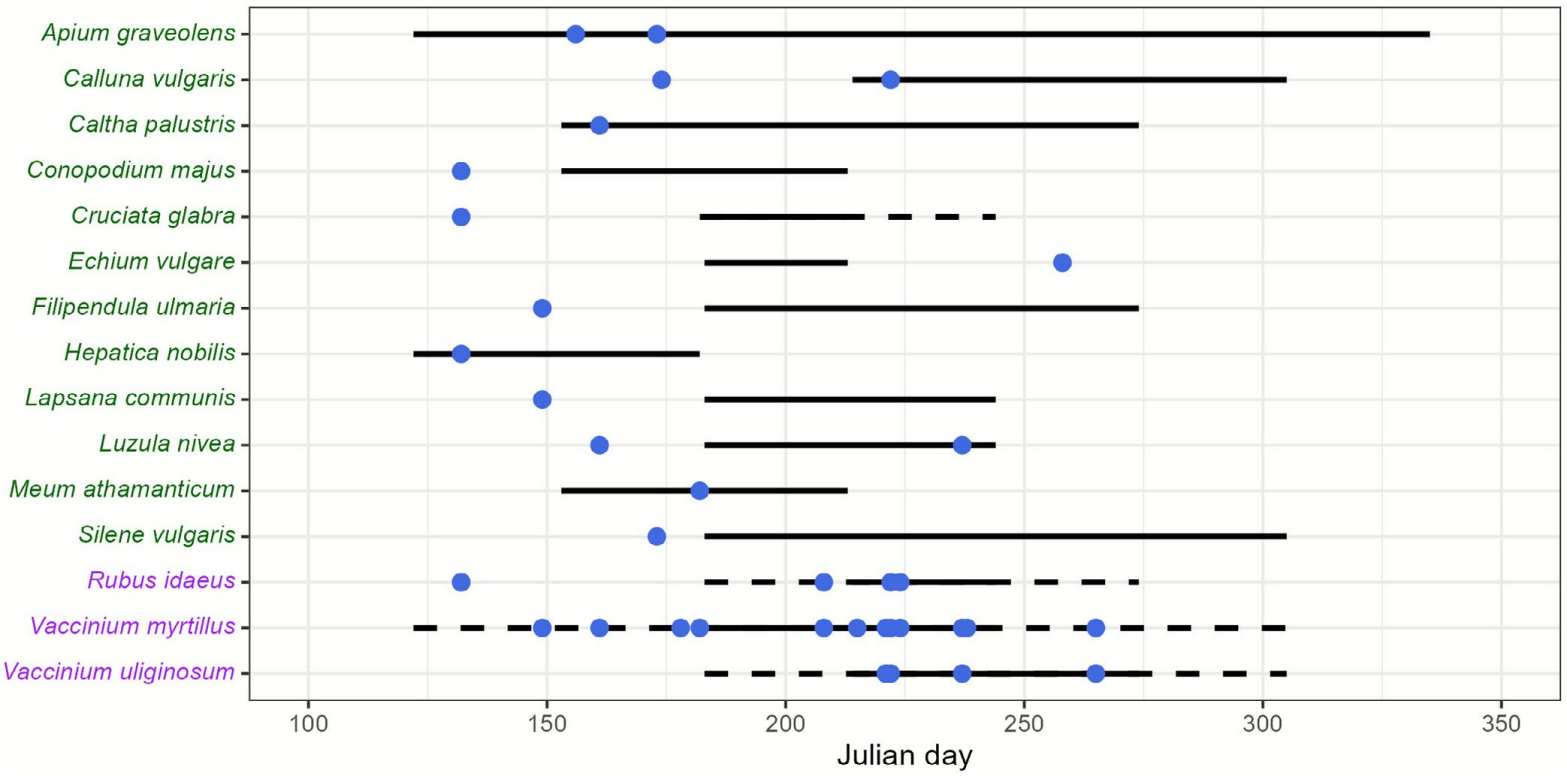
Occurrence of species (*Y* axis) identified in brown bear feces (blue dots: #4169E1) during the year (*X* axis in Julian day). For each species, the black solid line corresponds to the main fruiting period, while the black dotted line corresponds to the secondary fruiting periods; a species retrieved outside of these periods was not considered as dispersed. Dry‐fruited plants are written in green (#006400), fleshy‐fruited in purple (#800080).

**FIGURE 2:**
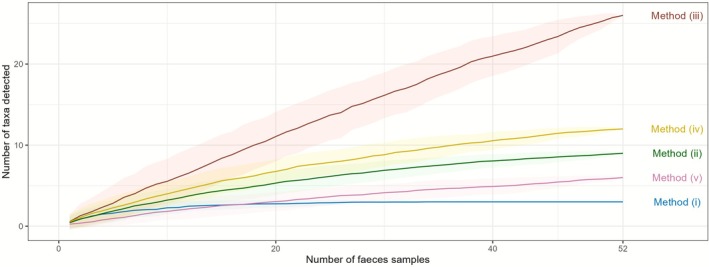
Taxa accumulation curves according to the method used (i.e., (i) fleshy fruits based on metabarcoding, (ii) plants during fruiting periods based on metabarcoding, (iii) seed extraction, (iv) disaggregated feces, and (v) aggregated feces).

**FIGURE 3:**
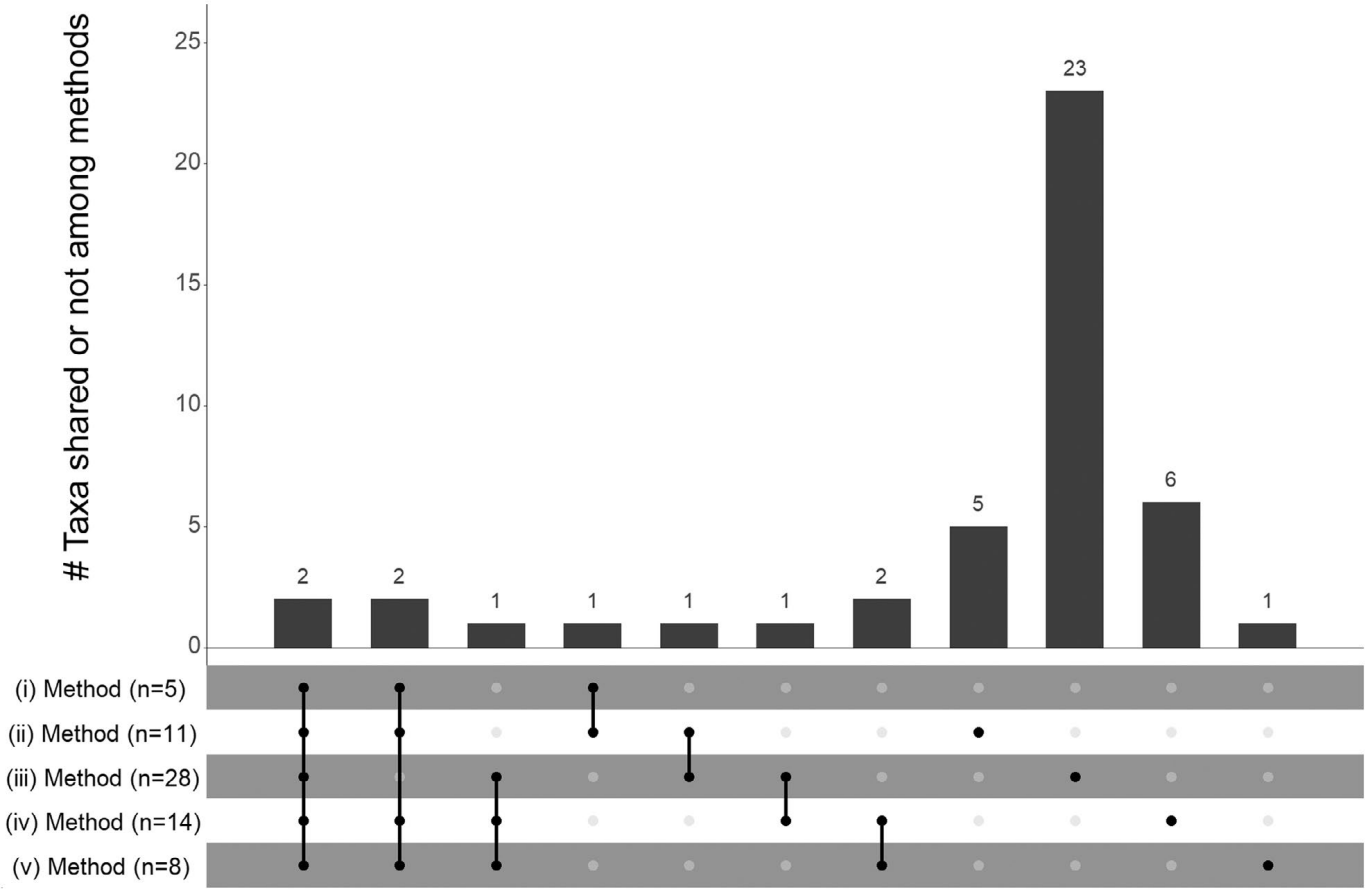
Number of taxa inferred per method (between brackets) and shared (connected black dots) or not among the five methods (i.e., (i) fleshy fruits based on metabarcoding, (ii) plants during fruiting periods based on metabarcoding, (iii) seed extraction, (iv) disaggregated feces, and (v) aggregated feces). The bar of the histogram represents the number of taxa shared by a unique combination of methods.

**FIGURE 4:**
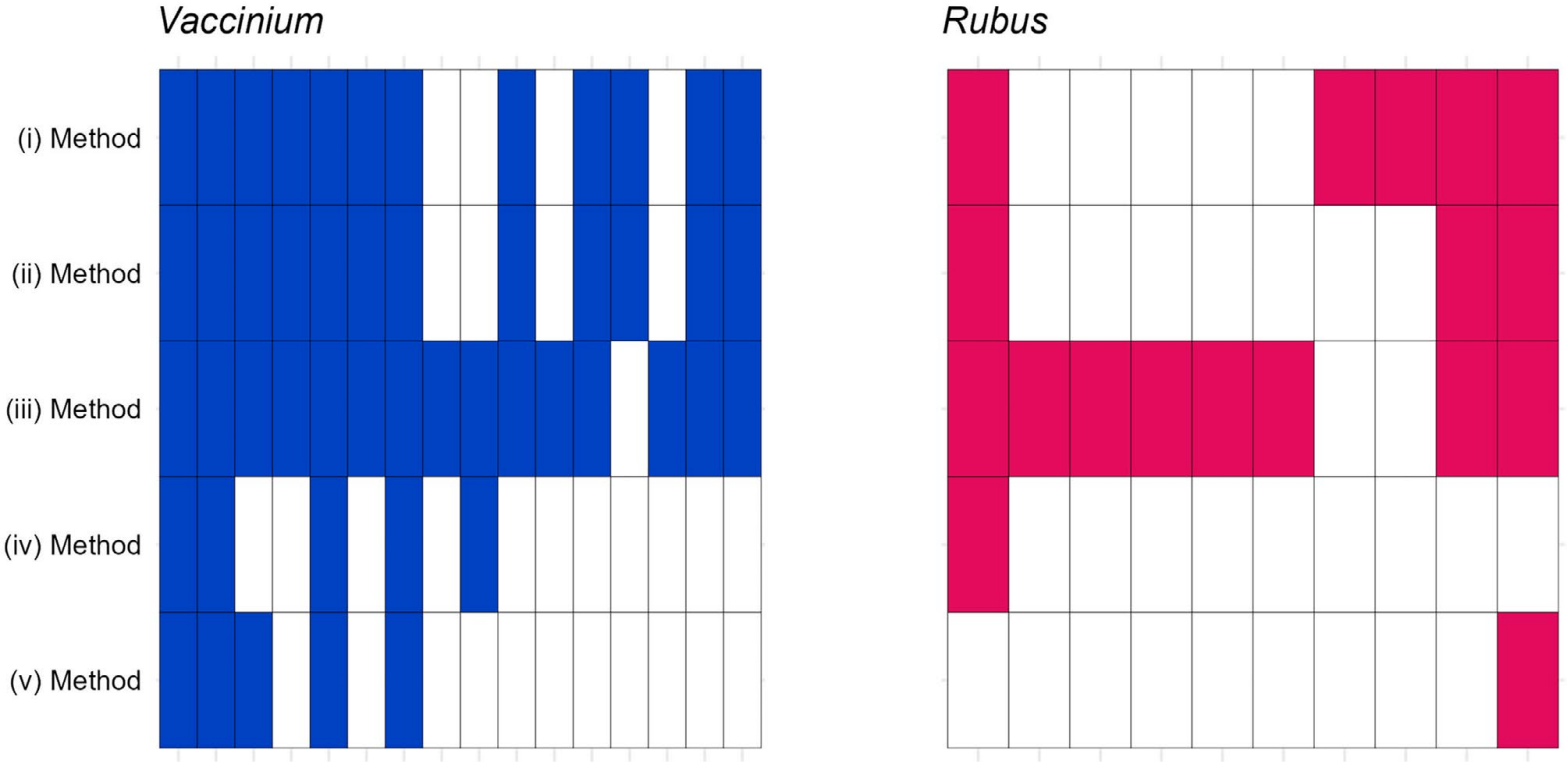
Detection of *Vaccinium* sp. and *Rubus* sp. (filled cell) per method (lines) and sample (columns). The methods were: (i) Fleshy fruits based on metabarcoding, (ii) plants during fruiting periods based on metabarcoding, (iii) seed extraction, (iv) disaggregated feces, and (v) aggregated feces.

**FIGURE 5:**
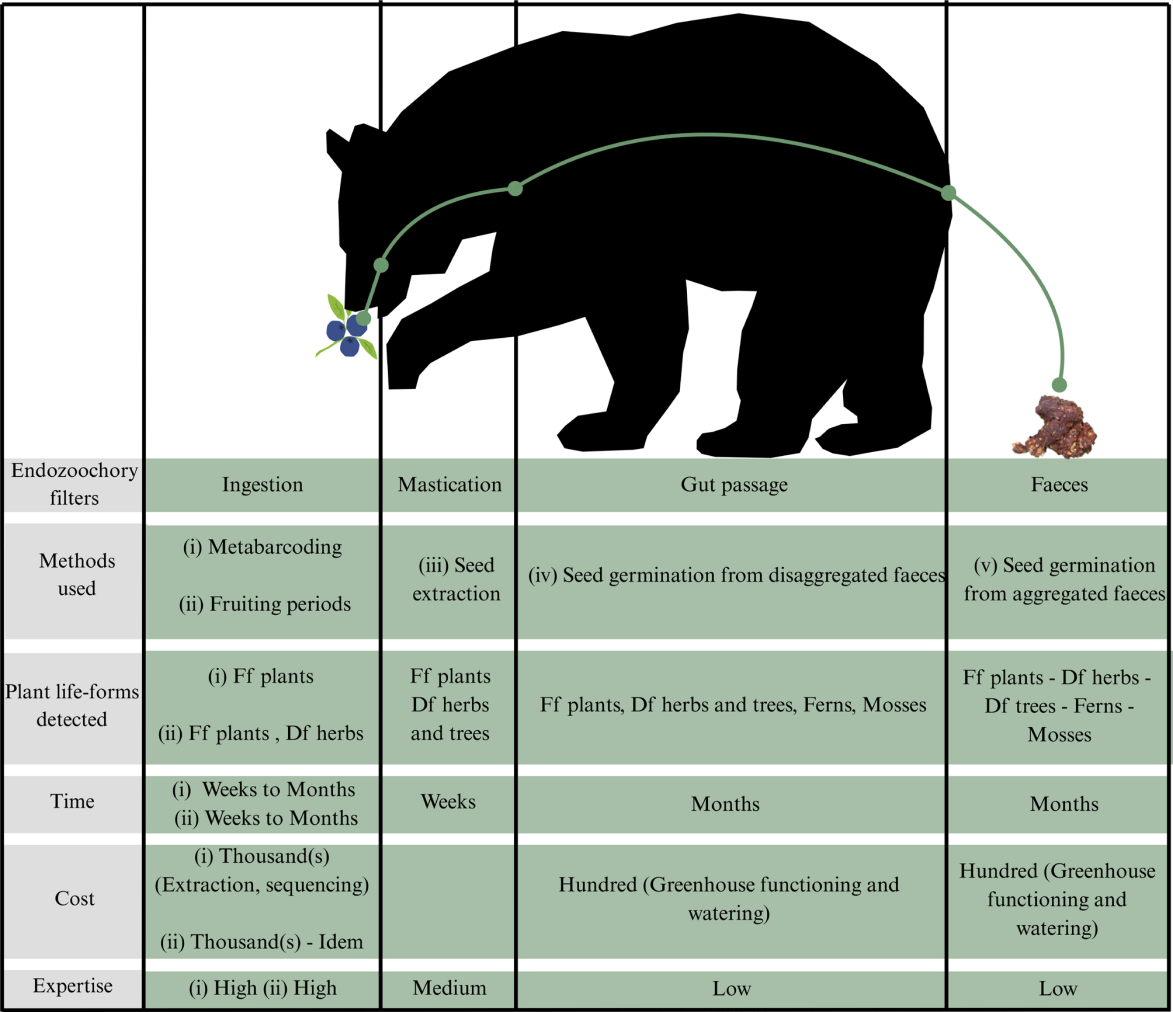
Chronology of the four different filters that diaspores face from the ingestion to the release in the fecal matrix, with the method(s) that handle each successive filter (i.e., (i) fleshy fruits based on metabarcoding, (ii) plants during fruiting periods based on metabarcoding, (iii) seed extraction, (iv) disaggregated feces and (v) aggregated feces.). We also represented the plant life‐forms detected (Ff plants = fleshy‐fruited plants; Df herbs = dry‐fruited herbs; Df trees = dry‐fruited trees; ferns and mosses), the time spent, the cost in euros, and the level of expertise, ranked from low to high, required for each method. The silhouette was obtained freely from Canva.com.

We performed all data analyses on R Studio (v.4.3.2). For the accumulation curves, we used the package *vegan* (v. 2.6.8) (Oksanen et al. 2007), and for the dissimilarity matrix test and turnover calculation, we respectively used *vegan* (v.2.6.8 2.6.8) (Oksanen et al. 2007) and betapart (v.1.6.1) (Baselga and Orme 2012) packages. We realized the Figures 1, 2, 3, 4 on R Studio and the Figure 5 using Canva.com.

## 3 Results

Based on metabarcoding of fecal eDNA, we identified 5919 OTUs (Operational Taxonomic Units), including 12,945,186 reads. Of these 5919 OTUs, 5785 were annotated to family level (nearly 100% of reads annotation, *n* = 12,939,806 reads), 624 to genus level (87.7% of reads annotation, *n* = 11,351,326 reads), and 297 to species level (1.4% of reads annotation, *n* = 175,219 reads). Thanks to the BLAST performed, we enhanced the taxonomic annotation of 13 OTUs (180,621 reads; including three species annotation: *Conopodium majus*, *Rubus idaeus* , and *Meum athamanticum*). Most of the annotations were hard mast bearing Fagaceae (2132 OTUs, 87.1% of annotations, *n* = 11,257,894 reads). We thus identified 53 different taxa, including 19 species, 23 genera, and 11 families (see details in Supporting Information).

### 3.1 Method (i): Metabarcoding and Fleshy Fruits Ingested

According to the metabarcoding results and the method (i), we identified three fleshy‐fruited plants considered as dispersed by endozoochory. Those plant species were scattered in 27% (14/52) of feces samples: *Vaccinium myrtillus* (9/52), *Vaccinium uliginosum* (4/52), and *Rubus idaeus* (5/52).

### 3.2 Method (ii): Metabarcoding and Plants Ingested During Fruiting Periods

Among the 19 plant species identified in the diet by metabarcoding, we found information on the fruiting period for 16 of them, permitting endozoochory investigation according to the method (ii) (data lacking for *Cynosurus cristatus* , *Salvia pratensis* , and *Nepeta cataria* ). Among those 16 species, we counted three fleshy‐fruited shrubs, 12 dry‐fruited herbaceous plants, and one hard‐mast woody plant (and not considered as dispersed). According to method (ii), we identified nine species consumed during their fruiting periods and inferred as dispersed by endozoochory (three fleshy‐fruited and six dry‐fruited plants). Those species were scattered in 29% of feces samples (15/52). Most of the fleshy‐fruited plants (90% of samples) were ingested during their fruiting period (main and secondary), whereas dry‐fruited plants (46% of samples) were ingested mostly outside their fruiting period (Figure 1).

### 3.3 Method (iii): Seed Extraction

We extracted a total of 1478 intact diaspores in 58% (30/52) of the feces samples, belonging to 27 different taxa, and—according to the method (iii)—these were considered as dispersed by endozoochory. We identified nine taxa at the species level, 13 at the genus level, and five at the family level. We found 66 diaspores from 15 different herbaceous plant taxa scattered in 28% (14/52) of the samples. We found at least 1405 diaspores from six fleshy‐fruited taxa ( *Ribes alpinum* ; *Rosa canina* ; *Rubus* sp.; *Vaccinium* sp.; *Sorbus* sp.; and *Juniperus* sp.) scattered in 37% (19/52) of the samples, and most of them were *Vaccinium* sp. (69%) and *Rubus* sp. (30%). We found only one diaspore from a dry‐fruited shrub ( *Calluna vulgaris* ). We finally retrieved six diaspores from four dry‐fruited woody plants scattered in 6% (3/52) of the samples, including two taxa producing small diaspores ( *Betula pendula* and *Betula pubescens* ) and two producing larger diaspores (*Abies* sp.; *Pinaceae* sp.).

### 3.4 Method (iv): Seed Germination From Disaggregated Feces

Those results were extracted from a previously published study (Pauly et al. 2024). Based on the germination experiment from disaggregated feces (*n* = 52), we detected plants (angiosperms, mosses and ferns) in 42% of feces samples (22/52), all considered as dispersed by endozoochory according to the method (iv). We observed the emergence of 164 angiosperm seedlings in 27% (15/52) of the samples and identified taxonomically 121 out of the 164 seedlings. We thus identified seven angiosperms, including one tree (*Betula* sp.), two fleshy‐fruited shrubs ( *Rubus idaeus* and *Vaccinium myrtillus* ), and four herbaceous taxa ( *Holcus lanatus* ; *Oxalis corniculata* ; *Persicaria hydropiper* ; and one *Asteraceae* sp.). *Vaccinium myrtillus* is the species that germinated the most (*n* = 5/52) and represented most of the seedlings identified (*n* = 114/121), whereas other taxa only counted one or two seedlings. We also observed 43 unidentified seedlings, but most of them were probably *Vaccinium* sp. (88%, *n* = 38/43). Furthermore, we detected one fern species ( *Dryopteris carthusiana* ) with three sprouts scattered in 6% (3/52) of samples and four mosses at the end of the monitoring ( *Leptodictyum riparium* ; *Ptychostomum dichotomum*; *Ceratodon purpureus* ; *Barbula convoluta* ) scattered in 17% (9/52) of samples.

As an alternative to the germination experiment from disaggregated feces, we performed Tetrazolium viability tests on 50 *Rubus* sp. and 50 *Vaccinium* sp. diaspores extracted and determined that 41% and 21% respectively of the diaspores tested were still viable. For non‐viable diaspores, we noted that the embryo was either aborted or intact but non‐viable.

### 3.5 Method (v): Seed Germination From Aggregated Feces

Those results were extracted from a previously published study (Pauly et al. 2024). Based on the germination experiment from aggregated feces (*n* = 52), we detected plants (angiosperms, mosses, and ferns) in 25% of feces samples (13/52), all considered to be dispersed by endozoochory according to the method (v). We observed the emergence of 85 angiosperm seedlings in 19% (*n* = 10) of the samples and identified taxonomically 41 out of the 85 seedlings. We thus identified three angiosperms, including one tree (*Betula* sp.) and two fleshy‐fruited shrubs ( *Rubus idaeus* and *Vaccinium myrtillus* ). *Vaccinium myrtillus* represented most of the germinations (*n* = 5) and seedlings (*n* = 39), whereas the two other taxa only counted one seedling. We also observed 44 unidentified seedlings, but most of them were probably *Vaccinium* sp. (93%, *n* = 41/45). Furthermore, we detected one fern species ( *Dryopteris carthusiana* ) with three sprouts in one sample (2%). We also identified two mosses at the end of the monitoring ( *Pseudoscleropodium purum* ; *Ceratodon purpureus* ) scattered in 6% (3/52) of samples.

### 3.6 Comparison of Methods

#### 3.6.1 Taxa Diversity

Among all the methods used, we identified potential plant dispersal in 78% of the samples (41/52) corresponding to 56 taxa. This included eight fleshy‐fruited shrub taxa ( *Rubus idaeus* ; *Vaccinium myrtillus* ; *Vaccinium uliginosum* ; *Ribes alpinum* ; *Rosa canina* ; *Juniperus* sp.; *Rubus* sp. and *Vaccinium* sp.), one fleshy‐fruited tree (*Sorbus* sp.), one dry‐fruited shrub ( *Calluna vulgaris* ), 35 dry‐fruited herbaceous plants (see details in Supporting Information), three dry‐fruited tree taxa ( *Betula pendula* ; *Betula pubescens; Betula* sp.), two hard‐mast tree taxa (*Pinaceae* sp., *Abies* sp.), one fern ( *Dryopteris carthusiana* ) and five mosses (*Barbula convoluta; Ceratodon purpureus; Leptodictyum riparium; Pseudoscleropodium purum; Ptychostomum dichotomum*).

Based on the observation of the accumulation curves on taxa detected per method, we observed that methods (i), (ii), (iv), and (v) seemed to stabilize at an asymptote and detected fewer than 15 taxa. Conversely, method (iii) detected more taxa in total and did not appear to reach an asymptote (Figure 2).

#### 3.6.2 Taxa Composition

Based on the plant inferred as dispersed by endozoochory in each method, we detected a significant difference in plant composition among methods based on the dissimilarity matrix analysis (Permanova, *F*‐value = 3.2604, *p* = 0.001, and *R* ^2^ = 0.13). The composition obtained with method (iii) was significantly different from all others, while no significant dissimilarity was detected between metabarcoding‐based methods (i) and (ii) or between germination‐based methods (iv) and (v). The method (v) was significantly different from metabarcoding‐based methods (i; ii), whereas the method (iv) was not different from metabarcoding‐based methods (Table 1 for comparison details). All feces combined, we calculated a high turnover (0.80) and nestedness (0.99) among methods.

**TABLE 1:** Statistical results based on permutation test on dissimilarity matrix per method.

Pairwise comparison	*F*‐value	*p*	*R* ^2^
Method (i)–Method (ii)^a^	0.9168	0.476	0.03284
Method (i)–Method (iii)	**5.583** ^2^	**0.001**	**0.11733**
Method (i)–Method (v)	**5.3008**	**0.001**	**0.15016**
Method (i)–Method (iv)	1.9892	0.085	0.08292
Method (ii)–Method (iii)	**4.5698**	**0.001**	**0.09607**
Method (ii)–Method (v)	**3.7675**	**0.001**	**0.10836**
Method (ii)–Method (iv)	1.2136	0.272	0.05012
Method (iii)–Method (v)	**3.2472**	**0.002**	**0.06594**
Method (iii)–Method (iv)	**2.0861**	**0.027**	**0.05204**
Method (v)–Method (iv)	1.2249	0.258	0.04499

*Note:* In bold: Statistically significant results.

^a^ Method (i) = metabarcoding and the fleshy‐fruited plants ingested; Method (ii) = metabarcoding and plants ingested during fruiting periods; Method (iii) = seed extraction; Method (iv) = seed germination from disaggregated feces; Method (v) = seed germination from aggregated feces.

In detail, two taxa were identified in all five methods (*Vaccinium* sp. and *Rubus* sp.) (Figure 3). Those two taxa were identified at the species level ( *Vaccinium myrtillus* and *Rubus idaeus* ) in methods (i), (ii), (iv), and (v), whereas method (iii) only reached the genus level. *Betula* sp. was shared by methods (iii), (iv), and (v). The moss species *Ceratodon purpureus* and the fern species *Dryopteris carthusiana* were exclusively identified in methods (iv) and (v). One species ( *Vaccinium uliginosum* ) was only identified in both methods (i) and (ii), another one ( *Calluna vulgaris* ) in both methods (ii) and (iii) only, and a last taxon (*Asteraceae* sp.) in both methods (iii) and (iv) only. In contrast, five taxa were found exclusively in method (ii), 23 exclusively in method (iii), six exclusively in method (iv), and one exclusively in method (v) (Figure 3).

Concerning *Vaccinium* sp. and *Rubus* sp. that were retrieved in each of the five methods, they were found respectively in 16 and 10 of the 52 fecal samples, with method (iii) inferring most of the dispersal events (80% and 94% of total detection, respectively, Figure 4). In two of the 52 fecal samples, we detected *Vaccinium* sp. in each method, which was not the case for *Rubus* sp. Method (ii) refined the results of Method (i) by removing two *Rubus* sp. occurrences. The methods (iv) and (v) provided the lowest detection, with 31% of the total detection for *Vaccinium* sp. and 10% of the total detection for *Rubus* sp. (Figure 4).

#### 3.6.3 Life Form Detection, Time, Cost and Expertise Required

All the methods allowed the inference of fleshy‐fruited plants. The methods (ii), (iii), (iv), and (v) allowed the inference of dry‐fruited plants, while only the methods (iv) and (v) permitted the inference of mosses and ferns (Figure 5). In terms of time, the methods (iii) were the fastest, whereas the germination‐based methods (iv) and (v) were the longest. In terms of cost, the methods (i) and (ii) were the most expensive (extraction and sequencing costs), whereas the methods (iii), (iv), and (v) were relatively cheap if we exclude available binocular or greenhouse (only functioning and watering costs). In terms of expertise, the methods (i) and (ii) required a high level of expertise (molecular tools and bioinformatics). In contrast, (iii), (iv), and (v) required only taxonomic expertise, which can be facilitated by the use of handbooks (Figure 5).

## 4 Discussion

In this study, we used brown bear feces to conduct five methods to infer endozoochorous dispersal according to the filters of endozoochory (ingestion, mastication, gut passage, feces). We highlighted the variability in the inference of endozoochory according to the combined effect of the methods and filters considered. First, contrary to our hypothesis, among the many different taxa inferred as dispersed by endozoochory, we found only a few taxa shared among methods. This difference could be related to the use of different sample masses from heterogeneous feces, which probably biased the comparison. However, we detected more overlap among methods for plant life forms detection, with fleshy‐fruited plants inferred in each method, dry‐fruited plants inferred in each method except method (i), and mosses and ferns inferred only in methods (iv) and (v). Although the greatest detection of plant life forms by germination‐based methods confirmed our hypothesis, the absence of moss and fern in metabarcoding‐based methods contradicted it. The absence of detection was probably related to the marker used (Taberlet et al. 2018). Furthermore, we observed a different number of taxa detected among the methods, with method (iii) resulting in the largest number of taxa inferred despite the use of only 5 g of feces material, which contradicted our hypothesis. This result may be related to the filters of endozoochory, as method (iii) did not consider the filters of gut passage and feces; some of the diaspores retrieved can be intact but non‐viable. On the contrary, it may be related to the requirements of diaspore germination not met for many diaspores, the low detectability of metabarcoding, or the intrinsic heterogeneous nature of the feces. Nevertheless, considering that method (v) was the only one to consider all the endozoochorous filters and inferred the greatest diversity of life forms, it appeared as the most reliable and complete method to infer endozoochory.

The metabarcoding‐based methods (i) and (ii) considered only the ingestion filter of the endozoochory process. Those methods were risky as they were based on the occurrence of a plant that was ingested either as a diaspore, shoot, leaf, or root, which may not indicate endozoochory at all. This is why the method (i) was only based on the consumption of fleshy‐fruited plants (endozoochorous syndrome; Van der Pijl 1982). The results for *Vaccinium* or *Rubus* show that the inferences of the method (i) were congruent with other methods most of the time. However, metabarcoding also missed other fleshy fruits retrieved in method (iii), including *Sorbus* sp. *Ribes* sp. *Rosa* sp. and *Juniperus* sp. We could explain this lack of detection by the incomplete reference sequence database or the length of the marker, which constrained taxonomic annotation. Based on metabarcoding, we also introduced a new method (ii) using fruiting period data of all plants retrieved in feces to infer potential diaspore ingestion. This method permitted going beyond the endozoochorous syndrome (Green et al. 2022) and allowed the investigation of dry‐fruited plant dispersal. We thus highlighted the dispersal by endozoochory of six dry‐fruited plants, among which one was inferred in other methods (but probably more if the entire feces were considered). Furthermore, using fruiting periods also refined the inference of fleshy‐fruited plants, by revealing the occurrence of *Rubus* in two feces outside its fruiting period, which is consistent with the absence of detection in methods (iii), (iv), and (v). This method could be improved with the enhancement of plant detection by metabarcoding, and more precise information on the fruiting period (e.g., fruiting periods of the year of feces collection). Although expensive, imperfect, and risky in terms of endozoochory inference, the use of metabarcoding may be used as an interesting tool for first approximation of endozoochory (especially for fleshy‐fruited plants) while providing detailed information on the animal diet (García‐Rodríguez, Selva, et al. 2021; Nawaz et al. 2019; Pauly et al. 2026).

Based on the method (iii), highlighting intact diaspores retrieved in feces, we inferred the largest number of taxa dispersed by endozoochory. Yet, this method may still miss tiny diaspore or spore dispersal as based on visual observation, even though it may be possible, but requires higher expertise and more time (e.g., Cázares and Trappe 1994). In the same way, the results may be overestimated due to intact but potentially non‐viable seeds. Moreover, visual identification encounters some limits for taxonomic annotation, as few morphological criteria exist to discriminate plant species (Cappers et al. 2006; Cappers and Bekker 2013). Dáttilo (2025) suggested that barcoding on extracted intact seeds can be a powerful tool to refine such results. Nevertheless, the method (iii) is less expensive than metabarcoding, faster than germination experiments (and not sensitive to seed germination requirements) and requires a medium level of expertise to identify diaspores. In this regard, the method (iii) was useful to infer endozoochory, despite the missed flora (mosses, ferns) and which did not consider the detrimental effect of the gut passage and the fecal matrix.

The germination‐based methods (v) and (vi) tested the viability of seeds through germination and thus considered the gut passage (Traveset and Verdú 2002). These methods permitted the detection of cryptic dispersal (mosses or ferns, Pauly et al. 2024). Such dispersal inference can also be due to contamination occurring between the defecation and the collection or from an airborne origin. We limited this contamination by the use of very fresh feces and that most species were not present near the greenhouse or in the controls (Pauly et al. 2024). For instance, endozoochorous dispersal of mosses and ferns has already been documented in other monogastric mammals respectively in flying fox *Pteropus conspicillatus* (Parsons et al. 2007) and wood moose *Apodemus sylvaticus* (Arosa et al. 2010). A recent review argued that endozoochory by large mammals could even be of ecological relevance for these plants (Brock 2025). Nevertheless, germination experiments are also sensitive to diaspore requirements for germination that may bias the results (Karimi et al. 2020). As an alternative to germination experiments from disaggregated feces, we performed the Tetrazolium test on *Rubus* and *Vaccinium* diaspores and quickly proved that most of the seeds were viable. On one hand, Tetrazolium tests may counter seed dormancy or other requirements and provide similar results to germination experiments at a faster rate. On the other hand, it requires important expertise, handling diaspores and potentially destroying them. This can induce bias and be time‐consuming according to the number of diaspores retrieved (Miller and Peters 2010; Soares et al. 2016). Therefore, the use of Tetrazolium offers an interesting but potentially riskier alternative to germination experiments.

Germination experiments from aggregated feces were the only method used that considered the four filters of endozoochory. Based on the original study (Pauly et al. 2024) aggregated feces represented a significant filter to germination compared to the disaggregated feces, as reported elsewhere (Enders and Vander Wall 2012; Mancilla‐Leytón et al. 2012; Jiménez‐Martín et al. 2025). To disaggregate brown bear feces in the Pyrénées, dung beetles seem to be the main biotic agent, while inducing secondary dispersal (Pauly, Vanpé, Roy, Sentilles, et al. 2025), but in Ursidae feces other agents can be observed, such as small mammals (e.g., Enders and Vander Wall 2012; Koike et al. 2012). Neglecting the fecal matrix filter may overestimate endozoochory but also overlook the crucial activity of fecal visitors and secondary dispersers in the plant dispersal process (Culot et al. 2015; Ishikawa 2011), all the more relevant for animals releasing large feces (Stricklan et al. 2020).

We acknowledge that our comparative approach was not perfect, as we used different sample masses from heterogeneous feces, which may explain the high turnover in taxa identified among methods. The use of similar masses, particularly for method (iii), could have revealed more similarities among the methods. In the same way, the passage in the freezer (−18°C) or the greenhouse monitoring may have biased germination ability and the results of methods (iv) and (v). Our study suggests that germination from aggregated feces provides a reliable method for inferring, but it remains biased according to germination requirements. In the same way, to better quantify the endozoochorous filters, feeding experiments with identified amounts of seeds, as previously done for target plants (Mancilla‐Leytón et al. 2011; Grande et al. 2013; Rubalcava‐Castillo et al. 2023; Tsunamoto et al. 2024) or animal materials (Lovas‐Kiss et al. 2024), may provide interesting results especially according to plant life forms. Furthermore, the methods used here are not the only ones for inferring endozoochory; others, such as the germination of seeds extracted on potting soil (e.g., Tavşanoğlu et al. 2021) or in Petri dishes (e.g., Lovas‐Kiss et al. 2023), can properly infer endozoochory but ignore the feces filter. Finally, diaspores' journey does not end with the release in the fecal matrix as the presence of secondary dispersers (Andresen and Urrea‐Galeano 2022) and the releasing site quality are both crucial to the germination and establishment success (Schupp et al. 2010).

This study encourages greater caution and nuance for the inference of endozoochory according to the method used and the filters considered. We draw particular attention to the plant life form detectability and confidence of inference. In this sense, the comparison among studies on endozoochory must look beyond simple methodological differences, but also consider the different filters tackled in these methods. Our results also suggest that the methods could complement each other to limit the biases of each. Finally, it may be interesting to analyze the most significant filters for different plants as performed in previous studies, in order to gain a more accurate understanding of the endozoochorous dispersal process and reduce part of the black box.

## Author Contributions

**Grégoire Pauly:** conceptualization (equal), data curation (lead), formal analysis (lead), investigation (lead), methodology (equal), project administration (equal), validation (equal), visualization (lead), writing – original draft (lead), writing – review and editing (equal). **Cécile Vanpé:** funding acquisition (equal), methodology (equal), project administration (equal), supervision (equal), validation (equal), visualization (supporting), writing – review and editing (equal). **Mélanie Roy:** data curation (supporting), formal analysis (supporting), funding acquisition (equal), investigation (supporting), methodology (equal), project administration (equal), supervision (equal), validation (equal), visualization (supporting), writing – review and editing (equal). **Yannis Clavier:** investigation (supporting), writing – review and editing (supporting). **Adélie Chevalier:** investigation (supporting). **Jérôme Sentilles:** resources (lead). **Tanguy Daufresne:** funding acquisition (equal), methodology (equal), project administration (equal), supervision (equal), validation (equal), writing – review and editing (supporting). **Christophe Baltzinger:** conceptualization (equal), data curation (supporting), formal analysis (supporting), funding acquisition (equal), investigation (supporting), methodology (equal), project administration (equal), supervision (equal), validation (equal), visualization (supporting), writing – review and editing (equal).

## Funding

This work was supported by Agreement N° OFB‐22‐1125 between ‘Office français de la biodiversité’ and INRAE and N° OFB.22.0773 between ‘Office français de la biodiversité’ and CNRS.

## Conflicts of Interest

The authors declare no conflicts of interest.

## Supporting information


**Data S1:** ece372589‐sup‐0001‐Supinfo1.csv.

**Data S2:** ece372589‐sup‐0002‐Supinfo2.csv.

**Data S3:** ece372589‐sup‐0003‐Supinfo3.csv.

**Data S4:** ece372589‐sup‐0004‐AppendixS1.docx.

## Data Availability

Data are available in Supporting Information.
