# Supplementary material for: Inferring Endozoochory From Ingestion to Germination Through Biological Filters: Brown Bear Feces as a Case Study
Source: Ecol Evol. 2025 Dec 2;15(12):e72589. doi: 10.1002/ece3.72589 (PMC12670293; doi:10.1002/ece3.72589)
Supplement: Supplementary file 4 — Data S4: ece372589‐sup‐0004‐AppendixS1.docx. [file ECE3-15-e72589-s001.docx]

# Supplementary

Appendix S1: Methods for faecal eDNA metabarcoding using trnL marker for plant detection.

## Metabarcoding analyses

*DNA extraction, amplification and sequencing*

DNA extraction and metabarcoding analyses were all performed by us at the Molecular Biology and Microbiology Technical facility of the Molecular Biology and Bioinformatics Department of the Centre de Recherche sur la Biodiversité et l'Environnement (CRBE) research laboratory. Molecular analyses were performed in 52 bear faecal samples in 2022 using plant markers. DNA extraction was performed using NucleoSpin Soil kit (Macherey Nagel) adapted protocol as described by [Taberlet et al., (2012b)](https://www.zotero.org/google-docs/?M9eI58), starting from a large amount of material, 15 g of feces were suspended in 30 mL of phosphate buffer. Extraction negative controls were performed to monitor contamination during DNA extractions.

We used the Sper02 markers to amplify plants. The 20 µL PCR consisted of 2X Master Mix AmpliTaq Gold (Fisher), 0,008X Bovine Serum Albumine, 0.25 µM forward primer and 0.25 µM reverse primer and 2 µL DNA extract. The PCR profile had an initial denaturation step of 10 min at 95 °C, followed by a few cycles of 30 seconds at 95 °C, 30 seconds at the annealing temperature, 1 min at 72°C and a final 7 min elongation at 72°C. The annealing temperature and number of cycles were different between primers. Three PCR replicates were performed for all samples. Four negative controls of extraction and PCR were included every 92 samples to monitor contamination during PCR preparation. We also included four positive controls of DNA samples. Libraries were performed with TruSeq DNA Nano kit (Illumina) following the manufacturer’s instructions. The sequencing was performed on a NovaSeq 6000 of Illumina in 2 x 250 bp.

*Bioinformatics*

Sequence analysis was performed using the *OBITools v1.2.11* [(Boyer et al., 2016)](https://www.zotero.org/google-docs/?1DIL8c) and the *sumaclust* [(Mercier et al., 2013)](https://www.zotero.org/google-docs/?AnFtGo) softwares, through a modified version of the *Snakemake v7.20.0* [(Mölder et al., 2021)](https://www.zotero.org/google-docs/?zFVlLl) pipeline of [Benoiston (2022)](https://www.zotero.org/google-docs/?s4jeG8) (a taxonomic annotation rule was added). Forward and reverse reads were aligned with the *illuminapairedend* command. Alignments with a score below 40 were filtered out. Sequences were then assigned to samples using the *ngsfilter* command, allowing 0 and 2 errors on the tags and primers respectively. Identical sequences were merged with the *obiuniq* command. Next, low quality sequences were filtered out, i.e. sequences with less than 1 read in the whole dataset, sequences containing ambiguous bases or that are shorter than expected (< 10 bp). The remaining sequences were clustered into Molecular Operational Taxonomic Units (MOTUs) with *sumaclust* using a similarity threshold of 97%. The most abundant sequence of each MOTU was considered as its representative sequence and its abundance corresponded to the sum of the abundance of its members. Taxonomic annotation was performed with the *ecotag* program of the *OBITools*, using a subset of the *Genbank* database (release 259) extracted with the *ecoPCR* program [(Bellemain et al., 2010; Ficetola et al., 2010)](https://www.zotero.org/google-docs/?gNaOgk). This subset was reduced to the Pyrenean plants to annotate plant sequences. The list of Pyrenean plants was extracted from FLORAPYR (<https://atlasflorapyrenaea.eu>, plants whose presence status was "probable mistake" were excluded). Maximum number of mismatches allowed per primer was three; the minimum-maximum length of the *in silico* amplified DNA fragment, excluding primers was 10-230 bp. The *‘metabaR’* package [(Zinger et al., 2021)](https://www.zotero.org/google-docs/?NBvn2R) of R (v.4.4.2) was used to minimize PCR/sequencing errors, contaminant sequences, tag-jumps and dysfunctional PCRs. MOTUs with maximum abundance in negative controls were considered as contaminants, and those with a similarity below 90% to the reference sequence were considered degraded sequences or chimeras and discarded. MOTUs not belonging to the targeted clade were excluded. Noise from tag-jumps was reduced by removing a MOTU in a given PCR product if its relative abundance was less than 0.03% of the total MOTU abundance in the entire dataset. PCR replicates with sequencing depth below specific thresholds (< 1,000), and those with poor reproducibility were removed. The final MOTU table was produced by summing the reads from PCR replicates originating from the same sample.

To refine the annotation obtained with *ecotag*, we performed a Blast search using online NCBI resources on all the MOTUs with at least 250 reads not annotated at least at gender level. We chose the annotation (gender or family level) when there was a taxa with the best E value, percent of identity, and query cover, but also based on their occurrence using local databases (FLORAPYR; <https://atlasflorapyrenaea.eu>).

References

[Bellemain, E., Carlsen, T., Brochmann, C., Coissac, E., Taberlet, P., & Kauserud, H. (2010). ITS as an environmental DNA barcode for fungi: An in silico approach reveals potential PCR biases. *BMC Microbiology*, *10*(1), 189. https://doi.org/10.1186/1471-2180-10-189](https://www.zotero.org/google-docs/?9eN4ym)

[Benoiston, A.-S. (2022). *AnneSoBen/obitools_workflow* (Version v1.0.2) [Computer software]. Zenodo. https://doi.org/10.5281/ZENODO.6676577](https://www.zotero.org/google-docs/?9eN4ym)

[Boyer, F., Mercier, C., Bonin, A., Le Bras, Y., Taberlet, P., & Coissac, E. (2016). obitools: A unix ‐inspired software package for DNA metabarcoding. *Molecular Ecology Resources*, *16*(1), 176–182. https://doi.org/10.1111/1755-0998.12428](https://www.zotero.org/google-docs/?9eN4ym)

[Ficetola, G. F., Coissac, E., Zundel, S., Riaz, T., Shehzad, W., Bessière, J., Taberlet, P., & Pompanon, F. (2010). An In silico approach for the evaluation of DNA barcodes. *BMC Genomics*, *11*(1), 434. https://doi.org/10.1186/1471-2164-11-434](https://www.zotero.org/google-docs/?9eN4ym)

[Mercier, C., Boyer, F., Bonin, A., & Coissac, E. (2013). SUMATRA and SUMACLUST: fast and exact comparison and clustering of sequences. *Programs and Abstracts of the SeqBio 2013 Workshop.*, 27–29.](https://www.zotero.org/google-docs/?9eN4ym)

[Mölder, F., Jablonski, K. P., Letcher, B., Hall, M. B., Tomkins-Tinch, C. H., Sochat, V., Forster, J., Lee, S., Twardziok, S. O., Kanitz, A., Wilm, A., Holtgrewe, M., Rahmann, S., Nahnsen, S., & Köster, J. (2021). Sustainable data analysis with Snakemake. *F1000Research*, *10*, 33. https://doi.org/10.12688/f1000research.29032.2](https://www.zotero.org/google-docs/?9eN4ym)

[Taberlet, P., Prud’Homme, S. M., Campione, E., Roy, J., Miquel, C., Shehzad, W., Gielly, L., Rioux, D., Choler, P., Clément, J.-C., Melodelima, C., Pompanon, F., & Coissac, E. (2012). Soil sampling and isolation of extracellular DNA from large amount of starting material suitable for metabarcoding studies. *Molecular Ecology*, *21*(8), 1816–1820. https://doi.org/10.1111/j.1365-294X.2011.05317.x](https://www.zotero.org/google-docs/?9eN4ym)

[Zinger, L., Lionnet, C., Benoiston, A., Donald, J., Mercier, C., & Boyer, F. (2021). metabaR: An r package for the evaluation and improvement of DNA metabarcoding data quality. *Methods in Ecology and Evolution*, *12*(4), 586–592. https://doi.org/10.1111/2041-210X.13552](https://www.zotero.org/google-docs/?9eN4ym)
